# A Helpful Tool or Another Barrier? Exploring Older Adults’ Perspectives on Patient Portals

**DOI:** 10.1093/geroni/igaf122.3871

**Published:** 2025-12-31

**Authors:** Chung Hyeon Jeong, BoRin Kim, Amy Tietjen

**Affiliations:** University of New Hampshire - eHealth readiness, Durham, New Hampshire, United States; University of New Hampshire, University of New Hampshire, New Hampshire, United States; University of New Hampshire, Durham, Massachusetts, United States

## Abstract

While patient portal use among older adults increased during the COVID-19 pandemic, their perceptions and experiences with these tools remain underexplored. The Unified Theory of Acceptance and Use of Technology (UTAUT) highlights perceived usefulness, effort expectancy, and social influence as predictors of technology adoption. This qualitative study examined how these constructs are reflected in older adults’ perceptions of patient portals in the post-pandemic context. In spring 2025, we conducted semi-structured Zoom interviews with 19 community-dwelling adults aged 65 and older who had visited a healthcare provider in the past six months. Eligible participants lived independently, had internet access, and no cognitive impairment. Thematic analysis generated three themes that both reflect and extend the UTAUT framework: (1) “Not for Us, But for Them”—participants reported low perceived usefulness, viewing portals as serving providers’ efficiency more than patient needs; (2) “Technology with Warmth”—older adults valued human connection in care and emphasized the importance of tailoring portals to their needs and readiness; and (3) “We Don’t Talk About It”—participants described limited social influence, noting that portals were rarely discussed among peers, challenging assumptions that peer endorsement strongly predicts adoption. These findings suggest that older adults’ portal use is shaped not only by functional barriers but also by emotional trust and limited social diffusion. To foster meaningful adoption, strategies should enhance perceived usefulness, incorporate relational and culturally sensitive design, and provide education that improves both relevance and usability.

